# Predictors of Volunteering Rates in the Local Authority Districts (LADs) of England: Neuroticism, Socioeconomic Grade, Trust, and Racial/Ethnic Diversity

**DOI:** 10.5964/ejop.17375

**Published:** 2026-02-27

**Authors:** Stewart J. H. McCann

**Affiliations:** 1 Department of Psychology, Cape Breton University, Sydney, Nova Scotia, Canada; Dublin City University, Dublin, Ireland

**Keywords:** volunteering, racial/ethnic diversity, homophily, trust, social cohesion, identity, prosocial behavior

## Abstract

**Objective**: The purpose of this study was to examine relations of formal volunteering (FV) rates to neuroticism, socioeconomic grade (SEG), trust of neighbors, and racial/ethnic diversity (RED) in 316 local authority districts (LADs) in England to determine the replicability of earlier research on the US states. **Method**: A national indicator dataset provided the percentage of adults who engaged in FV in each LAD during 2008. LAD neuroticism scores were based on internet responses of 386,375 UK residents to the Big Five Inventory. SEG was based on four occupation grades from the 2011 UK census. RED was based on 2011 UK census data and the premise that maximum diversity occurs when each racial/ethnic group has equal proportions. Based on 2009 to 2015 data, the neighbor trust variable indicates the percent of persons in each LAD who believe they can trust their neighbors. The present research employed correlation and multiple regression strategies. It also evaluated the impacts of spatial autocorrelation. **Results**: LAD FV rates correlated negatively with neuroticism and RED, and positively with SEG and trust. The most important finding is that the significant negative relation between RED and FV was rendered nonsignificant or was greatly reduced when trust was controlled in sequential multiple regressions. Adjusting for spatial autocorrelation or not, the results were *substantively* the same. **Conclusions**: These results essentially replicate those of two earlier analyses using US states. Findings strongly suggest that trust is a *mediator* between RED and FV. *Speculative* interpretations focus on relations between homophily, RED, and trust.

Formal volunteering (FV) is “any contribution of unpaid time to the activities of organizations or established entities” (Lee & Brudney, 2012, p. 160). Researching predictors of FV is important both economically and socially. FV boosts an economy through unpaid work hours, estimated to have been worth 187.7 billion dollars in the US in 2019 (Independent Sector, 2020). FV also has a substantial role in fostering democratic functioning, social bonds, and national and cultural identity (e.g., Craig et al., 2018; Hustinx et al., 2010; Putnam, 2000, 2007).

Regarding FV, McCann (2023) posed the following questions in research using the 50 states of the US as the analytic units and the mean of the 2003, 2004, and 2005 state FV rates as the criterion: Are FV rates related to levels of racial/ethnic diversity (RED)? Could trust be the operative feature in such a relationship? The results showed higher RED was associated with lower state FV rates. This association was consistent for 15 different FV rates — overall, White, non-White, male, female, married, unmarried, and eight different age variables. These relations also persisted with the explicit or implicit statistical control of several variables including state socioeconomic status (SES) and resident neuroticism. However, higher trust of neighbors (Social Capital Project, 2018) also was associated with higher FV rates *and* lower RED. Most importantly, controlling solely for trust eliminated RED as a significant predictor of each of the 15 FV rates.

The results of the McCann (2023) study also were conceptually replicated in an unpublished analysis of the 48 contiguous US states using more recent FV rates based on the mean for 2012, 2013, 2014, and 2015 (see McCann, 2026a). Explicitly controlling for neuroticism, socioeconomic status (SES), and trust in a multiple regression equation eliminated RED as a significant predictor of FV rates. Controlling only for neuroticism and SES in another equation resulted in RED still accounting for a significant 27.0% of the variance in FV rates. Furthermore, as in McCann (2023), controlling only for trust also eliminated RED as a significant predictor of FV rates.

The present research is a conceptual replication of the McCann (2023) and the unpublished analysis (see McCann, 2026a) using Local Authority Districts (LADs) in England as the analytic units. Despite the apparent societal benefits and personal rewards that can accrue from higher engagement in FV, not all LADs of England show the same levels of commitment to such service. For example, LAD FV rates ranged from 35.9% in West Devon to 14.0% in the city of Kingston upon Hull among 324 UK LADs in 2008 (Sutcliffe & Holt, 2011). Therefore, it would seem beneficial to have a fuller understanding of why there are such discrepancies in volunteering in these different geographic administrative divisions, and whether trust as a contributing factor in the previous studies of US states relates in the same way with LADs in England as the analytic units.

Along with the US state-level findings of McCann (2023) and the unpublished analysis (see McCann, 2026a), there is other research that has demonstrated the relations of neuroticism, SES, trust, and RED to FV. Most of these studies have used individuals as the analytic units. A very brief overview of examples of this research is presented in the following four subsections.

## Neuroticism and FV

A person high on the neuroticism dimension of the widely accepted nonpathological Big Five personality model (e.g., Costa & McCrae, 1995; Goldberg, 1990; John & Srivastava, 1999) has characteristics that may make FV less likely. For example, such a person tends to be anxious, nervous, self-conscious, easily agitated in tense situations, and generally unable to manage stress effectively. As well, there is evidence with individuals and US states as analytic units showing that higher neuroticism is associated with lower FV levels (e.g., Claxton-Oldfield & Banzen, 2010; Handy & Cnaan, 2007; King et al., 2015; McCann, 2017).

## SES and FV

Lower SES also is associated with lower levels of FV with individuals as the analytic units (e.g., Macchia & Whillans, 2021; Steptoe & Zaninotto, 2020; Wilson, 2000). This relation probably occurs because lower SES persons face more barriers to serving as volunteers such as limitations in income and other resources, including suitable knowledge and requisite skills (Musick & Wilson, 2008). Lower SES also has been related to lower US state FV rates (e.g., McCann, 2017, 2023).

## Trust and FV

Generalized trust refers to trust of persons external to family and friends. Where there is less trust, there is less community social interaction (Putnam, 2007) and less cooperation (Gächter et al., 2004). Where there is less opportunity for common purpose and cooperation, there is a weaker desire to participate in FV (Dinesen et al., 2020). Of course, trust *and* social interaction *and* cooperation are integral in most FV commitments. Researchers have found that generalized trust is positively associated with FV at the individual and state analytic levels (e.g., Dinesen et al., 2020; McCann, 2023; Miranti & Evans, 2019).

## RED and FV

Nesbit et al. (2020) have noted that RED “remains an underexplored topic in volunteering studies” (p. 919). RED relates negatively to social trust (e.g., Dinesen et al., 2020; Nesbit et al., 2020; Putnam, 2000, 2007). Higher RED in an area heightens the probability of exposure to those who are racially/ethnically different. Trust thrives on the perception of similarity of others to oneself because such perceived similarity “is an indicator of shared norms and other behaviour-regulating features relevant for trust” (Dinesen et al., 2020, p. 444).

The meta-analysis by Dinesen et al. (2020) found that trust in neighbors was strongest as a factor when the assessment of RED centered on “geographically bounded … residential contexts” broadly defined as “neighborhoods … municipalities, metropolitan areas, regions, and countries” (p. 443). Dinesen et al. (2020) suggest that “a plausible interpretation of the stronger relationship between ethnic diversity and trust in neighbors than for generalized social trust is that exposure to racially/ethnically dissimilar others is a stronger and more directly relevant cue for trust in neighbors than for trust in other people in general” (p. 452). Public and social media accounts also tend to make RED “much more visible, seemingly more local, and of more immediate concern” (McCann, 2023, p. 112). Such trust positively related to FV in the state-level research of McCann (2023) and in the later unpublished analysis (see McCann, 2026a).

McCann (2023) also speculated that principles based on mere exposure and homophily are fundamental to relations between RED, trust, and FV. The homophily principle recognizes that people have strong desires and tendencies to associate and interact with similar others (e.g., Alesina & La Ferrara, 2000; Haun & Over, 2015; Zhao, 2023). Homophily and trust have been positively related in various contexts (e.g., Ahlf et al., 2019; Evans & Wensley, 2009; Kim, 2015). Higher exposure to RED “implies a larger dose of negative cues regarding the trustworthiness of others in general” (Dinesen et al., 2020, p. 445). Lower trust is associated with less interpersonal contact and cooperation (Gächter et al., 2004; Putnam, 2007). Most FV activities depend upon trust, interpersonal contact, and cooperation. Consequently, lower trust is likely to result in less FV.

The focus of the current research is on the relation of RED to FV. The general picture painted here is that higher levels of RED in an area are associated with a lower likelihood of engaging in FV in that area. Although this account tends to attach negative connotations to RED, it is the widespread popular view of RED that it is beneficial in other circumstances. For example, many believe that it may enhance cognitive skills, critical thinking, and creativity in the classroom (e.g., American University, 2019), may improve problem solving, innovation, and productivity in the workplace (e.g., Mattina, 2025), and may reduce prejudice, increase empathy, provide opportunities for stronger social cohesion, and foster equity at the societal level (e.g., Ricee, 2023). It should be stressed that the current project is solely concerned with the empirical relation of RED to FV, and that any potentially negative connotations regarding RED engendered by the research should not influence perceptions of other relational contexts.

## The Present Study

The primary objective of the current study was to provide an initial cross-cultural test of the major findings of the McCann (2023) research and the unpublished analysis (see McCann, 2026a). The main hypothesis was that, although LAD FV rates may be negatively correlated with neuroticism and RED and positively correlated with socioeconomic grade (SEG) and trust, statistically controlling for trust of neighbors may eliminate any association between higher RED and FV rates. Such results would support the findings of McCann (2023) and the unpublished analysis and strongly suggest that trust mediates the relation between RED and FV in UK LADs as well as in US states.

The association between higher RED and lower trust has been found in both the US and the UK (e.g., Dinesen et al., 2020). However, Helbling et al. (2015) found that the relation is more likely to exist in countries demonstrating a stronger preoccupation with immigration issues. For example, Dinesen and Sonderskov (2018) noted that in the US “high levels of ethnic diversity paired with an idiosyncratic pattern of adverse race and ethnic relations has formed a national backdrop particularly conducive to conflictual local interactions eroding social trust” (ResearchGate version, p. 7), and “that political elites and their rhetoric play a role in connecting citizens’ experiences with diversity to their perception of the generalized other” (ResearchGate version, p. 8). These same factors also may be operative in England.

There also is evidence of adequate variability in FV rates (Sutcliffe & Holt, 2011) as well as sufficient differences in cultural, economic, and historical characteristics in the LADs of England to facilitate a replication of McCann (2023). For example, the LADs differ on the Big Five personality dimension of neuroticism (Rentfrow et al., 2015). As well, “the concept of a North–South divide, a line that dissects England, rendering the two halves economically, socially and culturally different, permeates English history” and shows clear differences in educational opportunities, poverty, wealth, employment, infrastructure, investment, transportation options, health, mortality rates, and political preferences (Burton, 2021). The seven regions of England (i.e., South West, South, South East, London, Midlands, North West, and North East) also differ markedly on social trust, including trust of others in general, of those met for the first time, and of those of another nationality (The Policy Institute, 2023). Furthermore, research shows that greater FV is associated with stronger “horizontal” social cohesion based on neighborliness, trust in others, and tolerance of people from other social groups, and that levels of such cohesion differ across the regions and LADs of England (Abrams et al., 2023). The LADs also have vastly different proportions of ethnic groups represented in their populations (Census, 2021). All of these categories of factors appear to be related to FV, thereby enabling a replication of the US findings. More specifically, it would seem possible to determine whether higher FV rates occur in LADS lower on neuroticism, higher on SEG, higher on trust, and lower in RED, and, whether the multivariable pattern of relations found with US state data also pertains to English LADs.

Cultural *similarities* between the US and the UK also suggest the possibility of successfully replicating the US results of McCann (2023) and the unpublished analysis (see McCann, 2026a) with English data. Although FV rates vary considerably across European nations (Gil-Lacruz et al., 2017), the rate was 24% in England in 2008 (Statista, 2024) compared to the rate of 26.4% in the US in 2008 (BLS, 2008). Therefore, the overall levels of FV in the US and England are relatively close. Researchers also have found that the association between higher ethnic diversity and lower trust may occur in both the US and the UK. For example, McCann (2023) found such a link in the US and Lymperopoulou et al. (2022) found that there was a negative association between ethnic diversity and trust in the UK. To the degree that there are cultural similarities between the US and the UK, it is reasonable that we should be somewhat more confident that the main findings of McCann (2023) will replicate in England.

However, although it is apparent that there are cultural similarities between the US and the UK, *differences* also exist that could be relevant to formal volunteering, ethnic diversity, and trust, as the following examples indicate. Americans are more likely to value and foster individualistic lifestyles while Britons tend to have a somewhat stronger focus on community and collectivistic responsibility (Harvey, 2023). Ideological polarization has been more pronounced in the US than the UK on cultural issues regarding the prioritizing of national customs and the importance of assimilating immigrants into the nation’s way of life (Silver, 2021). In a study of 13 countries including those as different as Canada, China, Netherlands, and India, the US was highest on social motivation for volunteering and England was the third lowest — only higher than Japan and Croatia (Grönlund et al., 2011). The US has a more diverse population speaking several different languages because it has a much longer and stronger history of immigration from many different countries, whereas English has long been the overwhelmingly predominant language commonly spoken in the UK (Harvey, 2023). The UK also has been demonstrated to be markedly higher than the US on social trust, including trust of others in general, those of a different social group, those in the neighborhood, those known personally, those met for the first time, and those of another nationality (The Policy Institute, 2023). Despite the many similarities between the two countries, these cultural differences between the US and the UK suggest that a successful cross-cultural replication of the general results pattern found by McCann (2023) and the unpublished analysis (see McCann, 2026a) was not a foregone conclusion.

It is important to know whether the US state-level relations also are found with the LADs of England as the analytic units. There are some studies using UK data that report relations of FV to neuroticism (e.g., Claxton-Oldfield et al., 2012), SEG (e.g., Sharp et al., 2023), trust (e.g., Storm, 2015), and RED (e.g., Hylton et al., 2019). Others report relations of neuroticism to SEG (e.g., Rentfrow et al., 2015), relations of SEG to trust (e.g., Janmaat, 2019), and RED (e.g., Pesta et al., 2023), and relations of trust to RED (e.g., Lymperopoulou et al., 2022). However, no existing research examines the interrelations of FV, neuroticism, SEG, trust, and RED in a multivariable framework with either individuals or LADs of England as the analytic units. The present study seeks to fill this void, at least at the LAD level of analysis.

The present study constitutes a cross-cultural test of the major thrust of the original study by McCann (2023). The study includes overall FV rates, resident neuroticism, SEG, trust of neighbors, and RED measures for each of 316 LADs in England. Variables are based on data collected between 2008 and 2015. Specifically, the following hypotheses are based directly on the results of McCann (2023) and the unpublished analysis (see McCann, 2026a):

1. Higher FV is associated with lower neuroticism.
2. Higher FV is associated with higher SEG.
3. Higher FV is associated with higher trust.
4. Higher FV is associated with lower RED.
5. Higher neuroticism is associated with lower SEG.
6. Higher neuroticism is associated with lower trust.
7. Neuroticism is not related to RED.
8. Higher SEG is associated with higher trust.
9. SEG is not related to RED.
10. Lower trust is associated with higher RED.
11. Controlling for trust eliminates the association between RED and FV.

## Method

### Measures

The FV rates in the present study pertain to 2008. The decision to choose 2008 was made solely on the basis of the availability of data for the individual LADs of England. Such FV data are not regularly collected and tabulated. The Sutcliffe and Holt (2011) data set presented a rare opportunity for the focus of the present research. Therefore, it was desirable for data on the other variables to be as close to 2008 as possible. The only available assessment of the neuroticism dimension of personality was based on data collected from 2009 to 2011. The SEG and RED variables were derived from data collected for the 2011 UK census. The LAD neighbor trust index developed from 2009 to 2015 data based on three different sources including the 2011 census offered the best opportunity for suitable LAD trust scores close to the target year of 2008. To use the best or only data available for each variable, it was necessary to accept assessments spanning the period from 2008 to 2015 but largely from 2009 to 2011.

To maintain a data matrix with no missing data, eight of the 324 LADs with FV rates had to be excluded. Six of those LADs (i.e., Bolsover, Christchurch, Havant, Winchester, Rother, and Richmondshire) were excluded because they lacked trust scores. Isle of Wight also was excluded because it is surrounded by water and therefore its border does not technically touch that of any other LAD. Borders must touch at some point to be considered a “neighbor” when conducting the spatial autocorrelation analysis used in this study. Hastings also was excluded because it only touches the border of Rother which had already been excluded because it lacked a trust score. These exclusions reduced the functional N to 316 LADs.

#### FV Rate

A national indicator dataset (N1 6) of the National Audit Office (Sutcliffe & Holt, 2011) provided the percentage of adults who engaged in FV in each of 324 LADs during 2008.

#### Neuroticism

Rentfrow et al. (2015) provided neuroticism T-scores for 380 UK LADs based on the responses of 386,375 residents in England, Scotland, and Wales to the Big Five Inventory (John & Srivastava, 1999) in an internet survey conducted from November of 2009 to April of 2011. They reported that the LAD samples were quite representative when compared to 2011 UK census data. For example, the number of LAD respondents correlated .84 with LAD population, the median age of LAD respondents and LAD populations correlated .79, and the number of White, Black, Asian, and Mixed ethnicity in LAD samples and in LAD populations correlated .95, .92, .93, and .84, respectively. Regarding reliability, their neuroticism variable has a Cronbach alpha of .83.

#### SEG

Four levels, or grades, of occupations form the basis for SEG (UK Geographics, 2014). The 2011 census (ONS, 2014) provided the percent of household reference persons aged 16 to 74 years falling into each of the four grades in each LAD. The creation of the SEG index for the present study involved the following steps. For each LAD, the percent for the highest, second highest, third highest, and lowest category were respectively multiplied by 4, 3, 2, and 1. These four transformed values added together and divided by 100 produced scores for the final SEG index for 316 LADs — the maximum number of English LADs with complete data for each of the four components.

#### RED

The 2011 UK census classified racial/ethnic groups according to 18 categories. ONS (2020) furnished the percentage of the population in each category in each of 324 English LADs. The sum of four different White categories formed a White population percent variable. The sum of five Black categories indicated the Black population percentage. The sum of five Asian categories served as the Asian population percent. The lone Arab category indicated the Arab population percent. Subtracting the sum of the White, Black, Asian, and Arab population percents from 100 created an “Other” population percent variable. Based on the 316 LADs used in the present study, the following procedure produced a composite RED index based on the theoretical view that maximum diversity occurs when each racial/ethnic group has equal proportions. The absolute percentage points a racial/ethnic category is from 20 percent indicate how far that category is from its equal proportions target. For each LAD, these five absolute percentages transformed to z scores and reversed through multiplication by -1, then summed and the result transformed to z scores, formed the RED index. Its Cronbach alpha is .90. Higher scores indicate greater RED. The McCann (2023) study and the later unpublished analysis (see McCann, 2026a) also used a RED index constructed in this manner.

#### Trust

The present study used a measure of neighborhood trust developed by Shorthouse et al. (2019) from data provided in the 2011 UK Census, 2009–2010 and 2010–2011 Citizenship Survey, and the 2015 Indices of Deprivation. This neighbor trust index indicates the percentage of persons in each of 319 English LADs who believe that they can trust their neighbors.

### Analytic Strategy

After computing descriptive statistics and Pearson correlations for the five variables in the main analyses, two sequential ordinary least squares (OLS) multiple regression equations were computed with LAD FV rate as the criterion. In Equation 1, neuroticism entered first, SEG entered second, and RED entered third. Neuroticism and SEG essentially served as statistical controls while the relation of RED to FV rates was evaluated. In Equation 2, trust entered third between SEG on the second step and RED on the fourth. This effectively evaluated the relation of RED to the FV rate with neuroticism, SEG, *and* trust statistically controlled. Of course, the order of entry in a multiple regression equation has an impact on the variance accounted for in the criterion by each predictor but has no impact on the size of the β regression coefficient produced for each predictor.

The underlying questions of the present study were, (1) whether RED is related to FV rates with neuroticism and SEG statistically controlled, and (2) whether RED is continues to be related to FV rates when neuroticism, SEG, *and* trust are all statistically controlled? The β coefficients provide the fundamental answers and do not change with different variable entry orders. The changes in the variance accounted for in FV rates provide useful additional information for a *specific* entry order but may produce different values for different entry orders. The entry of neuroticism before SEG in each equation was somewhat arbitrary, but research does show that neuroticism may be a more prominent predictor of volunteering rates than SES at the US state level of analysis (McCann, 2017).

Each equation also was screened for multicollinearity and spatial autocorrelation. SPSS (Version 29) performed all statistical computations except those for spatial autocorrelation which employed R software (R, 2022). The study used a statistical significance level of .05 throughout.

## Results

Table 1 displays the means, standard deviations, and Pearson correlations for the 316 LADs. Notably, FV rates correlated significantly with neuroticism (-.42), SEG (.41), trust (.64), and RED (-.35). Trust also correlated significantly with RED (-.75).

**Table 1:** Means, Standard Deviations, and Pearson Correlations for the 316 LADs

Variable	*M*	*SD*	1	2	3	4
1. Formal volunteering (FV) rate	23.81	4.56				
2. Neuroticism	50.04	9.21	-.42***			
3. SEG	2.53	0.23	.41***	-.52***		
4. Trust	0.51	0.17	.64***	-.32***	.30***	
5. Racial/ethnic diversity (RED)	0.00	1.00	-.35***	.09	.11	-.75***

*p < .05. **p < .01. ***p < .001. Two-tailed tests.

Table 2 shows the results of two multiple regression equations demonstrating the capacity of neuroticism, SEG, RED, and trust to predict LAD FV rates. The assumptions of multiple regression were generally met for each equation. For example, scatterplots indicated appropriate linearity, there was no autocorrelation in the residuals of the two equations as indicated by a Durbin-Watson value of 2.01 in the first and 2.03 in the second, there was no evidence of multicollinearity in either equation as shown by VIFs ranging from 1.04 to 3.35 which are well below the common threshold of 10 and even the more conservative threshold of 5, and Mahalanbois distance analysis showed no multivariate outliers. Also, normal predicted probability (P-P) plots indicated no significant departure from normality for residuals, and the scatterplot of the standardized residuals and the standardized predicted values showed acceptably even spread, indicating support for homoscedasticity. In addition, histograms showed normality of residuals and Q-Q plots of the standardized residuals also appeared normal, although the Kolmogorov-Smirnov test suggested some departure from normality (*p* = .040) for Equation 1 while the Shapiro-Wilk test did not. In contrast, both tests indicated normality for Equation 2.

**Table 2:** Multiple Regression Equations Demonstrating the Predictive Capacity of Neuroticism, SEG, RED, and Trust in Relation to LAD FV Rates

Equation	Step	Entry method	Predictors	*df*	Δ*R*^2^	*F*	β	*t*
1	1	forced	Neuroticism	1, 314	.173	65.61***	-.20	-3.76***
	2	forced	SEG	1, 313	.052	20.84***	.34	6.35***
	3	forced	RED	1, 312	.133	64.45***	-.37	-8.03***
2	1	forced	Neuroticism	1, 314	.173	65.61***	-.15	-3.15**
	2	forced	SEG	1, 313	.052	20.84***	.13	2.35*
	3	forced	Trust	1, 312	.250	148.57***	.64	8.56***
	4	forced	RED	1, 311	.005	3.02	.12	1.74

*p < .05. **p < .01. ***p < .001. Two-tailed tests.

In Equation 1, neuroticism entered on Step 1 accounted for 17.3% of the FV rate variance, *F*(1, 314) = 65.61, *p* < .001. SEG entered on Step 2 accounted for another 5.2%, *F*(1, 313) = 20.84, *p* < .001. RED entered on Step 3 accounted for a final 13.3%, *F*(1, 312) = 64.45, *p* < .001. The significant βs were -.20 for neuroticism, .34 for SEG, and -.37 for RED.

Equation 2 had the same form as Equation 1, but trust entered on Step 3 and RED entered on Step 4. Trust accounted for a further 25.0% of the FV variance, *F*(1, 312) = 148.57, *p* < .001. However, RED now could account for only a nonsignificant .5% of the FV rate variance, *F*(1, 311) = 3.02, *p* < .001. The significant βs were -.15 for neuroticism, .13 for SEG, and .64 for trust.

### Comparison of UK and US Results

Comparisons can be made between the multiple regression results using neuroticism, SES or SEG, trust, and RED in England and the US as predictors of FV rates. The present data for England show that controlling for neuroticism, SES, and trust eliminated RED as a predictor of LAD FV rates (see Table 2). Based on the unpublished analysis (see McCann, 2026a) of US state data, controlling for neuroticism, SES, and trust also eliminated RED as a significant predictor of FV rates (see Table 3). However, based on the McCann (2023) data, controlling for neuroticism, SES, and trust substantially reduced but did not eliminate the capacity of RED to predict overall FV rates (see Table 4). The data for the present study are openly available at McCann (2026b).

**Table 3:** Multiple Regression Equations Demonstrating the Predictive Capacity of Neuroticism, SES, RED, and Trust in Relation to State FV Rates in the Unpublished U.S. Analysis

Equation	Step	Entry method	Predictors	*df*	Δ*R*^2^	*F*	β	*t*
1	1	forced	Neuroticism	1, 46	.311	20.72***	-.52	-6.84***
	2	forced	SES	1, 45	.172	14.99***	.36	4.65***
	3	forced	RED	1, 44	.270	48.07***	-.52	-6.93***
2	1	forced	Neuroticism	1, 46	.311	20.72***	-.41	-5.24***
	2	forced	SES	1, 45	.172	14.99***	.21	2.48*
	3	forced	Trust	1, 44	.303	62.13***	.44	3.31**
	4	forced	RED	1, 43	.017	3.82	-.22	-1.95

*p < .05. **p < .01. ***p < .001. Two-tailed tests.

**Table 4:** Multiple Regression Equations Demonstrating the Predictive Capacity of Neuroticism, SES, RED, and Trust in Relation to State FV Rates in McCann (2023)

Equation	Step	Entry method	Predictors	*df*	Δ*R*^2^	*F*	β	*t*
1	1	forced	Neuroticism	1, 48	.294	19.99***	-.56	-7.19***
	2	forced	SES	1, 47	.059	4.28*	.23	2.92**
	3	forced	RED	1, 46	.391	70.03***	-.63	-8.37***
2	1	forced	Neuroticism	1, 48	.294	19.99***	-.45	-5.55***
	2	forced	SES	1, 47	.059	4.28*	.13	1.59
	3	forced	Trust	1, 46	.374	63.18***	.36	3.00**
	4	forced	RED	1, 45	.059	12.35***	-.38	-3.52***

*p < .05. **p < .01. ***p < .001. Two-tailed tests.

Another exploratory comparative approach dealt with the capacity of trust alone as a control to eliminate RED as a predictor of FV rates. McCann (2023) found that controlling solely for trust eliminated the relation of RED to state FV rates. RED alone accounted for 32.7% of the FV rate variance. With trust as the only control, this variance accounting capacity was reduced to a nonsignificant .3%. Similarly, the unpublished analysis based on more recent US data (see McCann, 2026a) found that RED alone accounted for 29.0% of the variance, but this was reduced to a nonsignificant .4% with trust controlled. The same analysis with the data for England showed that RED alone accounted for a significant 12.5% of the variance in FV rates. However, controlling for trust did not *eliminate* RED as a predictor but just substantially *reduced* its variance accounting capacity to a still significant 3.3%.

### Spatial Autocorrelation

Spatial autocorrelation (e.g., Anselin, 2003) exists when there is spatial clustering of similar or dissimilar values on a variable. Spatial clustering of similar values is referred to as positive spatial autocorrelation; spatial clustering of dissimilar values is referred to as negative spatial autocorrelation. Such structured spatial variation violates the assumption of independence of residuals when using OLS multiple regression. Significant positive or negative spatial autocorrelation can lead to biased regression coefficients and probability values. This potentially can present interpretive challenges for unadjusted OLS multiple regression results.

For the analysis of UK data, the two equations in Table 2 were tested for spatial autocorrelation using Moran’s I test for residuals (e.g., Anselin, 2003). To meet the requirements of the analysis using the R *haven, spdep,* and *spatialreg* packages (R Project, 2022), the five variables were standardized. A 316 x 316 Queen’s binary neighborhood matrix also recorded each LAD that touched another LAD at any point as a neighbor. Moran’s I analysis was based on a simultaneous block entry equation in R using the three and four variables included respectively in the two original equations.

Table 5 includes the spatial autocorrelation results for England. Moran’s I statistic standard deviate — the key decision criterion — was 11.30 (*p* < .001) for Equation 1 and 9.79 (*p* < .001) for Equation 2, showing that spatial autocorrelation existed. Lagrange multiplier diagnostics then indicated that a spatial error model (i.e., *errorsarlm* model in R) was most efficient to adjust for spatial autocorrelation.

**Table 5:** Spatial Autocorrelation Results for the Regression Equations for UK and US Comparisons

Database	Equation	Moran’s I^a^	Predictors	Original equation βs	Adjusted equation βs
Present UK analysis^b^	1	11.30***	Neuroticism	-.20***	-.14***
			SEG	.34***	.44***
			RED	-.37***	-.12*
	2	9.79***	Neuroticism	-.15**	-.13**
			SEG	.13*	.29***
			Trust	.64***	.42***
			RED	.12	.12
Unpublished US analysis	1	.08	Neuroticism	-.52***	N/A
			SES	.36***	N/A
			RED	-.52***	N/A
	2	.64	Neuroticism	-.41***	N/A
			SES	.21*	N/A
			Trust	.44**	N/A
			RED	-.22	N/A
McCann (2023) US analysis	1	.87	Neuroticism	-.56***	N/A
			SES	.23**	N/A
			RED	-.63***	N/A
	2	.67	Neuroticism	-.45***	N/A
			SES	.13	N/A
			Trust	.36**	N/A
			RED	-.38***	N/A
Present UK analysis	A	11.08***	RED	-.35***	-.20***
	B	8.64***	Trust	.84***	.68***
			RED	.27***	.22**
Unpublished US analysis	A	2.87**	RED	-.54***	-.44***
	B	1.52	Trust	.87***	N/A
			RED	.08	N/A
McCann (2023) US analysis	A	3.29***	RED	-.61***	-.49***
	B	1.98*	Trust	.79***	.70***
			RED	-.02	-.01

*Note.* ^a^Moran’s I statistic standard deviate. ^b^These two equations used a spatial error model for adjustments while all other equations used a spatial lag model.

*p < .05. **p < .01. ***p < .001. Two-tailed tests.

Table 5 shows that the pattern of significant β coefficients for the spatial error adjusted equations closely resembled the pattern for the original equations. Adjusting for spatial autocorrelation or not, the βs for the two equations were *substantively* the same. They did vary somewhat in magnitude, but not in relational direction or in whether they were statistically significant or not. In other words, one would reach the same basic conclusions whether spatial autocorrelation was controlled or not. Therefore, it is reasonable to interpret this *pattern* of the results for England according to the original OLS equations.

Spatial autocorrelation analysis also tested the five regression equations developed in the comparisons of UK and US results and adjusted βs where applicable (see Table 5). In contrast to the UK analysis, the 3-variable and 4-variable equations for the unpublished US analysis and the McCann (2023) US analysis showed no evidence of spatial autocorrelation. The 1-variable A equations with only RED as a predictor did have significant spatial autocorrelation using each of the three databases. However, the adjusted βs did not differ substantively from any of the original βs. The 2-variable B equations with trust and RED as predictors also had significant spatial autocorrelation in the present UK analysis and in the McCann (2023) analysis. However, again the adjusted βs did not differ substantively from the original βs. Therefore, it is reasonable to interpret all patterns of results for England and the US according to the original OLS equations.

## Discussion

The findings of McCann (2023) and the later previously unpublished analysis (McCann, 2026a) were successfully replicated with data for the 316 English LADs. The first 10 hypotheses were supported by the Pearson correlations. As expected, FV rates correlated positively with SEG and trust of neighbors, and negatively with neuroticism and RED. Neuroticism correlated negatively with SEG and trust. SEG correlated positively with trust. Trust correlated negatively with RED. Also as expected, RED did not correlate with neuroticism or SEG. These correlations substantively paralleled the US results found in McCann (2023) and in the unpublished analysis.

The 11^th^ hypothesis that “controlling for trust eliminates the association between RED and FV” also received support from the planned analysis using the 3-and-4-variable approach. Controlling for neuroticism and SEG did not eliminate RED as a predictor of FV rates, but adding trust as a third control did so. This pattern of results also emerged in the unpublished analysis. However, such an analysis applied to the McCann (2023) data did not wholly support this outcome. In the 4-variable equation, SES did not have a significant β weight, and trust as a third control substantially reduced but did not eliminate the predictive capacity of RED.

However, the UK support for the 11^th^ hypothesis was somewhat qualified by the exploratory 1-and-2-variable analysis in which controlling *only* for trust greatly *reduced* but did not entirely *eliminate* the capacity of RED to predict LAD FV rates. In other words, controlling for neuroticism, SEG, and trust *eliminated* RED as a predictor of LAD FV rates, but controlling for neuroticism alone only *reduced* the predictive capacity of RED in this UK context. In contrast, the US results based on McCann (2023) and the unpublished analysis showed that RED was *eliminated* with trust as the sole control variable.

The results strongly suggest that trust serves as a *mediator* between RED and FV. However, formal mediation analysis generally is not recommended when the researcher possesses only cross-sectional data without clear temporal sequencing (e.g., Fairchild & McDaniel, 2017; O’Laughlin et al., 2018; Shrout, 2011). Nevertheless, Shrout (2011) does suggest that mediation analysis with cross-sectional data might be somewhat informative if the research context is one “in which there are well-founded theories that describe the causal direction of the processes, and for which the interpretation of the cross-sectional measures is informative about the temporal process” (p. 857).

The present study assumed that the temporal sequence in the indirect path in the present study is from RED to trust to FV, but there has not been sufficient argument for this chain of events to justify mediation analysis in the current analytic strategy according to the stated qualifications of Shrout (2011). Nevertheless, in an exploratory fashion, hypothetically assuming that one could argue successfully for such a clear temporal sequence, an application of the Sobel Test of Mediation (Preacher & Leonardelli, 2025) did suggest that trust may mediate the relation between RED and FV. Equations including neuroticism and SEG as statistical controls yielded a Sobel Test statistic of -8.12, a standard error of .28, and a probability value less than .001. Equations without these two statistical controls produced a Sobel Test statistic of -11.10, a standard error of .26, and a probability value less than .001.

Geographical psychology researchers (e.g., McCann, 2023; Rentfrow et al., 2008; Rentfrow & Jokela, 2016) are aware that aggregate level results *may* or *may not* successfully extrapolate from one level to the other. There are dangers of committing the compositional fallacy (e.g., Pettigrew, 1997) or the ecological fallacy (e.g., Robinson, 1950) if such extrapolations are based purely on *assumptions* rather than empirical evidence at *both* levels. Nevertheless, despite cautions, it is often useful to put forth judicious aggregate-level *speculations* based on cross-level extrapolative rationale grounded in individual-level dynamics and vice versa (Rentfrow & Gosling, 2021). The *speculation* here is that the LAD-level results stem from the aggregation of corresponding individual-level relations.

The major thrust of the findings with English LADs is the same as in McCann (2023) and the unpublished analysis (McCann, 2026a) for US states: Statistically controlling trust eliminates or drastically reduces the relation between RED and FV. The *speculation* here is that the principle of homophily is at the root of the link between RED and trust. Perceived racial/ethnic differences tend to signal dissimilarity of values, attitudes, beliefs, and behaviors. High RED fosters distrust. In turn, lower trust is not conducive to interpersonal contact and cooperation. FV then suffers because it depends to a large extent on personal interaction and cooperative efforts with others — often initially strangers. Individual-level studies also support the plausibility of these *speculations*. For example, researchers have found that trust relates positively to homophily (Ahlf et al., 2019) and to FV (Miranti & Evans, 2019); that homophily relates positively to FV (Wiertz, 2016); and that RED relates negatively to trust (Putnam, 2007), homophily (Bacharach et al., 2005), and FV (Putnam, 2007).

Although relations between trust, homophily, and trust may indeed provide a plausible explanation for the current results, it remains possible that the interrelations of other variables might also furnish viable alternative interpretations. Variables such as the individual FV rates of different racial/ethnic groups, the differences in racial/ethnic population percents, the homeland cultural norms regarding FV of those not born in the UK, the specific barriers to FV faced by particular racial/ethnic groups, and the relative availability of FV opportunities for different racial/ethnic groups may be implicated to some degree in the relation between RED and FV rates. Of course, theoretically articulating and testing how such variables might afford an explanation of the association between higher RED and lower FV rates is well beyond the scope of the current study. At present, the explanation focusing on trust, homophily, and RED seems most viable, elegant, and parsimonious.

Regarding spatial autocorrelation, perhaps one could have somewhat anticipated that spatial clustering would be likely for the UK data. Relatively short distances even between more distant LADs fairly readily facilitates the geographic dispersion of ethnic groups in England. More closely adjacent LADs also make it more likely that clusters of LADs may share beliefs and attitudes on a multitude of issues and events, including FV and RED. Nonetheless, as previously noted, incorporating spatial autocorrelation adjustments into the UK analysis produced no *substantive* difference compared to the original OLS multiple regression coefficients. Similarly, when spatial autocorrelation was detected in the US data, there was no *substantive* difference between spatially adjusted and original OLS results.

Should we expect that the results patterns for the US (McCann, 2023; McCann, 2026a) and England regarding trust, RED, and FV demonstrated in the present study also applies to other nations? Both the US and England have a predominantly White population steeped in individualism where FV is an established part of the social fabric (e.g., Kemmelmeier et al., 2006). As well, Dinesen and Sonderskov (2018) noted that the US has a history of “high levels of ethnic diversity paired with an idiosyncratic pattern of adverse race and ethnic relations” (p. 7) and thereby may be particularly susceptible to the erosion of trust. They also assert that the rhetoric of elite politicians and their followers influences citizens to associate their experiences with RED to their perception of others in general and this subsequently leads to lower trust. England may provide a somewhat similar context. Dinesen and Sonderskov (2018) also pointed out that the relation between RED and trust may be weaker in other Western countries than in the US. However, England likely is among the relative exceptions.

Helbling et al. (2015) suggest that a strong negative relation between RED and trust is only likely in nations having a relatively high preoccupation with issues of immigration. However, there has been a tremendous increase recently in immigration in many countries, including England. Diversity, equity, and inclusion (DEI) movements also have proliferated, generally elevating political turbulence and polarization. Increases in RED undoubtedly have prompted commensurate concern with immigration issues making it more probable that many other countries will likely show the pattern of relation between trust, RED, and FV found in the present research and that of McCann (2023) and the previously unpublished analysis (see McCann, 2026a).

### Limitations and Issues

The current UK study used a cross-sectional approach with statistical analyses directly or indirectly based on correlations. Therefore the “correlation-not-causation” rule applies. One must confine inferences regarding causality to *speculation.* None of the reported relations can serve as *empirical evidence* of *causal* relations, although some may be consistent with such *speculated causal* dynamics.

One also should exercise caution when interpreting the *independent* contributions of the predictors. The forced entry order was neuroticism, SEG, trust, and then RED in the 4-predictor multiple regressions. This order does not have an impact on the size and statistical significance of the regression coefficients, which are stable indications of the magnitude and direction of the relation of each predictor to the FV criterion with all other predictors controlled. However, the variance accounted for in FV rates by each predictor *is* contingent upon entry order.

Readers also should note that the formula for the RED measure in the present research uses the official UK census categories for “ethnic” classifications. Of course, an obvious shortcoming is that they fail to fully distinguish the variety of cultural identities and countries of ancestral origin that are inherent in categories such as Other Black, Other Asian, or Arab. Nor do they explicitly describe ethnic characteristics or differences in ethnic identities within or between these official categories. Both race and ethnicity are social constructs (e.g., Afridi & Warmington, 2009; Sandefur et al., 2004), but ethnicity more accurately — albeit imperfectly — reflects cultural differences and ancestral nations of origin. The present research conceptually centers on the overall RED of a geographical administrative analytic unit but is reliant on the UK census data as collected and reported. However, nothing in the current study pertains to specific races or ethnic groups. It also is noteworthy that census respondents had the opportunity to make their own choices according to the category option with which they had the strongest *self-identification.*

### Implications for Application

The s*peculation* here, as in McCann’s (2023) article, is that the principle of homophily is at the root of the relation between RED and interpersonal trust. Therefore, policy and promotion efforts should aim at the positive modification of several targets — FV, social cohesion, trust, racial/ethnic identities, national identity, and the experience of homophily. Efforts to encourage FV *or* greater social cohesion may lead to an increase in both. As well, efforts to improve levels of trust or counter the dynamics of homophily are likely to strengthen social cohesion, increase FV among all racial/ethnic subpopulations, and contribute to a heightened, more positive, sense of racial/ethnic and/or national identity. However, as Baldassarri and Abascal (2020) have noted, any attempts directed at increasing volunteering in ethnic subgroups should proceed with knowledge of the fact that volunteering opportunities must not be available only for those in exclusive networks but must be appropriate and inviting for ethnic subgroups with full appreciation of their specific economic, social, political, institutional, and cultural realities.

### Future Research

The present line of inquiry suggests several promising research avenues stemming from gaps in the literature that have become more apparent given the current findings. For example, the present postulations effectively cast trust as a mediator between RED and FV. Therefore, it would be beneficial to use appropriate longitudinal and perhaps experimental or quasi-experimental research to test the validity of this assumption. Many researchers consider that fruitful mediation analysis depends upon causative temporal sequence, which one cannot determine from cross-sectional data (e.g., Fairchild & McDaniel, 2017; O’Laughlin et al., 2018; Shrout, 2011).

Establishing more firmly that the aggregate-level relations found in the present research stem from the additive accumulation of corresponding relational dynamics at the individual level of functioning constitutes another worthy research path. Multilevel modeling procedures (e.g., Hox & Roberts, 2010) could determine the dependence or lack thereof between individual-level and aggregate-level relations. However, such a procedure would require researchers to assess each variable for each person in large representative samples from each geographical unit serving as a case in their analysis. This is a tall, but ultimately feasible, order.

A final example of a potential research direction based on the present line of study could involve examination of the relation of trust to FV rates within specific ethnic communities. For instance, existing US data show White, Black, and Hispanic ethnic groups vary markedly in their trust of neighbors (e.g., Gao, 2016). They also differ widely in their tendency to engage in FV (e.g., U.S. Bureau of Labor Statistics, 2016). To determine why this is so is a worthwhile research goal. The present UK analysis and the two US analyses used only a composite RED index.

### Conclusion

The present research showed that trust, RED, and FV are related in essentially the same manner in English LADs as in US states. Not only is FV related positively to trust and negatively to RED, controlling for trust eliminates or substantially reduces the relation between FV rates and RED. The ongoing worldwide population distribution reset brings with it potential promise and peril regarding issues associated with RED and national assimilation. One general impact of immigration appears to be higher distrust of others outside immediate family and friendship circles, and this appears to lead to lower interest in serving formally as a volunteer. This is unfortunate because FV makes an economic contribution, supports democratic functioning, facilitates social bonds, and builds and supports both national and ethnic cultural identity (e.g., Craig et al., 2018; Hustinx et al., 2010; Putnam, 2000, 2007; Warfield, 2016).

Like McCann’s (2023) research, the present study was conducted in the sensitive spirit of Putnam (2007) who recognized “both the threat and the promise of scientific research on the relation of diversity to social cohesion” (McCann, 2023, p. 100). Although the inherent issues may seem to some extent controversial, empirical research should continue efforts to disentangle the truths and falsehoods surrounding the impacts of increases in immigration in different nations — perhaps especially those focused on matters of wider social interaction and community involvement. Greater personal comfort levels with both national and ethnic identities could be the paramount benefit. That also could eventually contribute to lower intranational and international dissension, and greater acceptance, peace, and harmony.

## Supplementary Materials

**Table:** 

Type of supplementary materials	Availability/Access
Data	
England FV data 241217.	McCann (2026b)
Variable Key for England FV data 241217.	McCann (2026b)
Code	
No code was provided.	—
Material	
No study material available.	—
Study/Analysis preregistration	
The study was not preregistered.	—
Other	
Unpublished analysis on US volunteering rates.	McCann (2026a)

## Data Availability

The data for the present study are openly available at McCann (2026b).
